# The night is dark and full of hesitation. The latest step in the legal trajectory of assisted suicide in Italy: through tentative judicial openings, bioethical contradictions and the Parliament’s deafness

**DOI:** 10.3389/fphar.2025.1520136

**Published:** 2025-12-09

**Authors:** Paola Frati, Marco Albore, Eleonora Micaela Ertola, Sossio Del Prete, Letizia Sorace, Raffaele La Russa, Giorgio Bolino

**Affiliations:** 1 Department of Anatomical, Histological, Medical, Legal and Locomotor Apparatus Sciences, Faculty of Pharmacy and Medicine, Sapienza University of Rome, Rome, Italy; 2 Department of Clinical Medicine, Public Health, Life and Environmental Sciences, University of L'Aquila, L'Aquila, Abruzzo, Italy

**Keywords:** medical aid in dying, voluntary assisted death, assisted suicide, end-of-life, right to self-determination, life-sustaining treatments, constitutional legitimacy

## Abstract

The legalization of voluntary assisted dying, or physician-assisted suicide (AS), is gaining momentum as the recognition of a patient’s right to self-determination becomes increasingly integral to medical practice, even in cases involving extreme decisions, such as the cessation of therapy. In Italy, even Law no. 219/2017 already permits to refuse any medical intervention, including life-support treatment (LTS) and with the explicit aim of dying, with no possibility of hindering that choice. Subsequently, two landmark rulings by the Constitutional Court (ordinance no. 207/2018 and judgment no. 242/2019) marked a pivotal moment in legal, bioethical, and clinical discourse, fostering a shift toward a new care paradigm. Nevertheless, these developments remain tentative, reflecting the ongoing legislative gap in this domain. One of the most pressing issues is the restriction of AS to only patients who are dependent on LST thus scotomising a huge base of terminally ill sufferers. Several other significant judicial decisions have also shaped this area, including the Court of Assizes of Massa’s ruling on 27/07/2020, the recent order no. 32/2024 by the GIP of Florence, and the latest Constitutional Court’s 18/07/2024 ruling no. 135, which reaffirmed the previous set-up but also strengthened the validity of the albeit vague, extensive definition of LST formulated in 2020. We also discussed latest opinion of Italian National Committee of Bioethics regarding the definitional boundaries of LST. This article critically examines the concept of LST from clinical, bioethical, and legal perspectives, revealing its inconsistencies as a prerequisite for access to voluntary assistance in dying.

## 1 Introduction

The relentless technical advancement of modern medicine and the increasingly central role of the patient’s right to self-determination are generating complex bioethical-legal scenarios that require the ability of legislative systems to adapt in order to guarantee truly adequate solutions to society’s new priorities (Sallnow et al., 2022). In fact, the expansion of the tutelage of health self-determination has led to a profound redefinition of the sacrality and inalienability of life, supporting the autonomy of personal choice in all phases of existence by legitimising drastic decisions both in the initial (or even only potential) phases (Savulescu, 2002) and final stages (van Wijngaarden et al., 2015; Braun, 2023). Within certain limits, hence, it is now acceptable to dispose of own life, as an individual right, even renouncing it, and this can only presuppose the personal right to judge own existence not worth living.

In Italy, this vision was incarnated legislatively with Law No. 219 of 2017 which grants patients the right to make decisions about their own care, including the option to discontinue active treatment and life-sustaining treatments (LST), even if these had been planned in advance with the explicit aim of dying, with no possibility of hindering that choice (Di Fazio et al., 2021). The law ensures that patients may choose to resort solely to pain management (which can include continuous deep sedation, if necessary) and be guided through to the end of life. This choice is deemed compatible with constitutionally protected rights and values (Ciliberti et al., 2018). The main keystone of this progressive revolution in favour of the individual’s decision-making autonomy is clearly linked to the concept of informed consent, which, particularly in Italy, has contributed to settle secular bioethical debates in other spheres of ways of one’s own body disposition.

In addition to the established options for refusing medical treatments, there have been two significant rulings from the Italian Constitutional Court (CC) on the subject of physician-assisted suicide (PAS): Ordinance No. 207/2018 and Sentence No. 242/2019. These decisions marked a critical shift, not only legally but also from bioethical and clinical perspectives, signaling a substantial change in the caregiving paradigm (Turillazzi et al., 2021; Delbon et al., 2021; Cupelli, 2020).

However, these legal developments seem modest given the ongoing regulatory gaps in Italy, and they also reveal certain critical issues (Ricci et al., 2022; Marrone et al., 2022). Notably, the requirement for dependency on life-sustaining treatments (DLST) to access PAS is unique globally (Mroz et al., 2021; Jox, 2023; Emanuel et al., 2016; Dyer et al., 2015).

This issue was previously addressed by the Assize Court of Massa (judgment 27.7.2020) (Corte D’Assise di Massa, 2020), which advocated for a broader interpretation of LST. More recently, the preliminary investigation judge (*Giudice per le Indagini Preliminari,* GIP) of Florence referred the matter to the CC regarding the constitutionality of the DLST requirement in relation to the legal implications of PAS (Gazzetta Ufficiale, 2024).

On 18 July 2024, the CC released Sentence No. 135/2024, which rejected the GIP’s queries but affirmed the broad interpretation of the DLST requirement proposed by the Court of Massa in 2020. This interpretation stands in contrast to the majority opinion of the Italian National Bioethics Committee (CNB), which had suggested a more restrictive definition of DLST (National Bioethics Committ ee CNB, 2023).

The normative guidance provided by the Court rests largely on the application of Law No. 219/2017, most notably in Judgment No. 242/2019. Unless and until the legislature intervenes, this guidance remains the key reference point for assessing the legality of PAS. The Court also considered recent rulings from the European Court of Human Rights (ECtHR) on Hungarian case law, which, while allowing member countries significant decision-making autonomy, recognized the role of legal safeguards like DLST in preventing abuse (Author anonymous, 2024). Despite these important clarifications, there remains an ongoing climate of uncertainty surrounding end-of-life issues both internationally and within Italy, where there is a lack of a clear and specific national law on the subject and the jurisprudential references regarding end-of-life care remain the Constitutional Court’s sentences.

## 2 The trajectory of Italian jurisprudence on PAS: how did we get to this point?

### 2.1 Judgment no. 242/2019 of the Constitutional Court

As widely known among the Italian population, the case in exam involved a patient (nicknamed “DJ Fabo”) suffering from total and irreversible paralysis, immobilized and next to blind, though with preserved intellectual faculties, following a severe traffic accident. He was mechanically ventilated through endotracheal intubation (EI), fed via a percutaneous endoscopic gastrostomy (PEG), and required permanent bladder catheterization and assistance for bowel evacuation. Subsequently, with the help of activists and the staff of “Dignitas”, in February 2017, he resorted to the assisted suicide procedure in Switzerland by biting a device that autonomously triggered the injection of a lethal substance (Sodium Pentobarbital). The activist then voluntarily turned himself in to law enforcement in Milan. This event marked the beginning of the criminal prosecution which, in light of sentence no. 242/2019 of the CC (preceded by Ordinance No. 207 of 2018), led to his acquittal by the Court of Assizes of Milan on the grounds that “*the fact does not subsist*” (Montanari, 2019).

The Court’s ordinance does not aim to define the legal status of suicide; rather, the arguments fall within an intermediate position, neither adhering to the thesis of the structural illegality of suicidal conduct nor to the opposite conception of the right to suicide under Article 2 of the Italian Constitution, which would lead to the unconstitutionality of Article 580 of the Italian Criminal Code (ICC) (Constitutional Court Press Office, 2018).

Judgment No. 242/2019 by the Constitutional Court (following Order No. 207/2018) identified a constitutional issue with Article 580, Paragraph 2, of ICC, which addresses the crime of aiding and abetting suicide. The Court found that this provision does not adequately exclude from criminal liability individuals who assist in the suicide of a person who, despite being kept alive by LST, is suffering from an irreversible illness and intolerable physical *or* psychological pain, provided that the person is fully capable of making free and informed decisions. This assistance is permissible as long as these conditions have been verified by the National Health Service and after consulting a territorially competent ethics committee.

These four conditions^1^ were distilled from the case under review which, due to its absolute exceptionality, represents an extreme case.

According to the Court, in cases like that of DJ Fabo, the protective needs underlying the criminalization of physician-assisted suicide lose their solidity and may be called into question. The Court’s line of reasoning is rooted in the legislative framework set forth by Law No. 219 of 2017, which becomes a crucial reference point in its argumentation: “*Indeed, while the cardinal importance of the value of life does not exclude the obligation to respect the patient’s decision to end their own existence through the withdrawal of medical treatment—even when this requires active involvement, at least from a naturalistic perspective, by third parties (such as the disconnection or deactivation of a medical device, accompanied by the administration of deep continuous sedation and pain management)—there is no reason why the same value should serve as an absolute, criminally enforced barrier to granting the patient’s request for assistance in avoiding a slower, unwanted progression, perceived as contrary to their idea of a dignified death, following the aforementioned withdrawal of life-sustaining measures*.”

Following the Court’s reasoning, a case such as that of DJ Fabo involves a patient who would not be able to die “with dignity” through a “mere” refusal of therapy because his life-sustaining treatments are not among those of which the suspension would immediately result in death, but rather could lead to an unnatural and unwanted prolongation of life, often in great pain, for several days.

The Court identified the solution to this controversy, while awaiting parliamentary intervention, in an already established “*reference point within the system*” found in the “*framework of Articles 1 and 2 of Law No. 219 of 2017*”. In the Court’s view, the procedure outlined in Law No. 219 of 2017 appears to be the most suitable to address the “regulatory needs” already highlighted in Ordinance No. 207.

According to the CC, when a patient has the right to request the cessation of LST under Law No. 219 of 2017, with the explicit intention of dying, this right should also apply in cases like that of DJ Fabo. Consequently, refusal of therapies with lethal effects and PAS are considered equivalent in terms of constitutionally protected rights, both seen as legitimate ways to end life; however, the Court highlighted that there is a significant distinction between PAS and ‘*dying with dignity*’ through the mere refusal of treatments. In fact, the suspension of LST does not lead to immediate death but rather results in an extended and often painful existence, which can also affect the patient’s relatives. The central issue in Judgment No. 242/2019 is the absence of a detailed definition of the DLST, since the Court only referenced artificial ventilation, hydration, or feeding, focusing on the specifics of the case rather than providing a comprehensive definition. On the other hand, the Court could not have acted otherwise, since a ruling of the Constitutional Court must necessarily refer to the specific case under examination.

### 2.2 Judgment of the Assize Court of Massa in 2020

The judgment of the Assize Court of Massa on 27 July 2020 was an initial implementation of the right recognized by the CC in 2019. The case concerned D. T., a patient suffering from progressive multiple sclerosis. On 13 April 2017, an intravenous infusion was administered that, through a mechanism activated by the patient himself, injected the drug necessary to induce death. The crux of the matter in this case is the DLST. This is because, in the present case, the patient suffering from multiple sclerosis was not subjected to LSTs such as forced feeding and hydration, artificial respiration, and similar measures. His life went on with unbearable pain despite the absence of these treatments, although he was receiving other forms of support, and he was still able to ‘even’ breathe and eat independently.

However, upon closer inspection, D. T. could be considered dependent on two forms of life support.

He was undergoing pharmacological therapy aimed at pain management, which had reached maximum dosages: any increase, repeatedly requested by the patient, would lead to a drug overdose, while a reduction would result, in addition to unbearable pain exacerbation, in a worsening respiratory insufficiency and a hastening of the disease progression leading to death. Additionally, he was no longer autonomous as the paralysis of the intestinal muscles made daily manual evacuations essential, initially performed by healthcare providers and later, once the technique was learned, by his mother. Without these maneuvers, intestinal perforation and subsequent death would have occurred.

In this case, the judges determined that the condition of DLST includes any form of medical support that, if withdrawn, would lead to the patient’s death, even if not immediate. This broader definition encompasses scenarios where continued survival depends not only on medical equipment but also on ongoing pharmacological treatments or other forms of external healthcare, such as manual bowel evacuations or bladder catheterization. If a person relies on external assistance—whether from individuals or medical devices—to meet essential needs and thus to continue living, the condition required by the CC is considered fulfilled.

However, it is worthwhile to highlight that in patients who are irreversibly and intolerably ill, a general reliance on external aid might be presumed. As a result, the DLST requirement could lose its function as a selective evaluative criterion. The judges’ use of analogy in this context may appear to undermine the specific criteria established by the CC, rather than merely offering a broader interpretation.

### 2.3 Ordinance no. 32/2024 of the Florence GIP

The lack of a precise definition of DLST has led to interpretative challenges concerning constitutional mandates, as evidenced by the recent Order No. 32/2024 from the Florence GIP.17 The judge in this case rejected a request to dismiss charges of aiding suicide, arguing that the presence of the DLST requirement was only marginally identifiable and disputing the extensive interpretation of this criterion. The judge consequently referred a new question to the CC regarding the legitimacy of the DLST requirement.

That being said, in the absence of the penal coverage provided by the Court in 2019, the conduct of the individuals under investigation had to be considered an external action that, according to the standard criteria for determining causality, stands as a necessary antecedent to the death^2^.

The ordinance referring to the CC concerns the assistance provided by activists in December 2022 to a 44-year-old patient suffering from multiple sclerosis, to facilitate access to medically physician-assisted suicide in Switzerland through oral self-administration of the drug (using the right upper limb, still functioning). According to the ordinance, there was significant progression of the demyelination process typical of the disease, resulting in a worsening of the patient’s living conditions: initial walking difficulties, followed by the need for a wheelchair, and eventually being unable to move from his bed, with a near-total immobilization of the upper limbs.

However, the GIP founds these conditions insufficient to meet the requirement for DLST, although a somewhat “expansive” interpretation of the Court’s decision could apply given the necessary external interventions for fulfilling daily physiological needs resulting from irreversible bed confinement. The GIP thus challenges this reinterpretation of DLST, which could include all cases where “*the patient’s survival depends directly on others […], whether they be things or people*.”

The GIP applies a different and more restrictive assessment of the DLST requirement established by Judgment No. 242/19 of the Constitutional Court, which based its declaration of illegitimacy on Law No. 219/2017, specifically concerning *medical* treatments. The criminal prohibition of assistance in suicide established by the Constitutional Court would be unreasonable only in situations where the legitimacy of “*the patient ending their own existence through the withdrawal of medical treatments—even when this requires active conduct, at least from a naturalistic perspective, by third parties (such as the disconnection or deactivation of a medical device, accompanied by the administration of deep continuous sedation and pain management*)” is already recognized (under Law No. 219/2017). (Paragraph 2.2.3 of Order no. 32/2024 of the Florence GIP).

The conclusion is that the exemption from criminal liability for “aid to die” can only be justified in the context of medical treatments specifically, and not for all forms of “aid to live”.

These arguments are not entirely convincing, not least because they seem to represent a form of precautionary subtlety, intended to provide, at least partially, doctrinal support to what, in the opinion of the author, is the ultimate and genuine purpose of the ordinance: the pursuit of the long-desired legislative resolutions or, alternatively, an expansion of the current judicial safeguards by streamlining the nebulous requirement of dependence on treatments.

Should the need for assistance to eat, hydrate, perform physiological needs, take medication, or use technical aids considered essential for controlling one’s pathologies or functional alterations, even if not carried out by specialized healthcare personnel (though requiring technical-professional prescription or preparation), not be reasonably ascribed to a vital need for external support? What is the boundary, if any, within which a lifesaving, curative, or supportive act can qualify as medical? Is the ‘medical’ denomination strictly fundamental for these indispensable actions?

Furthermore, in Law No. 219/2017, the characteristics that determine the ‘medical’ nature of a treatment are not clearly detailed. Indeed, Article 1, paragraph 5 of Law No. 219/2017 governs the possibility of renouncing any treatments regardless of a terminal condition, with a completely different rationale: the universal self-determination of the patient at any level of healthcare management (diagnosis, therapies, interventions, assistance) or procedural invasiveness. Furthermore, this allows for the discontinuation, omission, or premature renunciation of any treatment, with explicit lethal effects, even in patients with a favorable prognosis and expected to live. According to Law No. 219/2017, such treatments need not involve conditions with the severity and irreversibility of the tetralogy indicated by the Court, as the assessment of therapeutic disproportion (Kon et al., 2016; Kitzinger and Kitzinger, 2017), according to the patient’s judgment, is broader and not subject to judicial review. In the complex realm of end-of-life care, it therefore seems inappropriate to apply, even with decisive imperativeness, a requirement that fails to find a clear definition or precise placement in this field, as it was conceived for entirely different and broader purposes (patient autonomy). Instead, this is a prerequisite for a positive ‘*request for assistance in the self-delivery of death’*: we are at the boundaries of the *right to die*.

### 2.4 The National Committee of Bioethics (CNB)

In the meantime, due to the constant interpretative difficulties in the processes of examining PAS requests stemming from the absence of clear guidelines on LST, the National Committee of Bioethics (CNB) has recently been called to express its opinion 18. A controversial majority opinion (18 members out of 32, with divergences even among them) claimed that LST should be interpreted as procedures that meet the following characteristics:

- They must address conditions that threaten life within a short or very short period (when it is not a matter of mere *support*, but an actual *replacement* of a vital function that the body is no longer capable to autonomously ensure);
- They must employ advanced technologies and specialized procedures, and can involve significant invasiveness and continuity over time, and therefore are not to be confused with a life-saving treatment or drug (for example, adrenaline for anaphylactic shock);
- Their suspension results in immediate or otherwise rapid fatal consequences, depending on the type of treatment and the clinical conditions of the patient.

According to this thesis, completely antithetical to the minority opinion, the legal-normative logic of the DLST constraint is to delimit—with a so-called ‘objective’ method—the area of facilitation for PAS to prevent possible abuses; although, just a few lines earlier, the lack of a shared definition in both legal and, especially, medical-scientific fields was acknowledged. So, where is this supposed objectivity?

Indeed, despite the already well-known definitional uncertainty, the opinion delineates the boundaries of LST—as opposed to those defined as *ordinary*—by the immediate proximity to death in the absence of them, that is, the sudden cessation of the vital functions that they replaced upon their interruption. The cornerstone, therefore, is the *replacement* of vital functions and not just their *support*. Where the clear difference between the two lies, not in semantic terms but rather in clinical-health terms, is not well understood or, at least, its utility is not apparent in the complex universe of end-of-life care. Moreover, one of the major implicit paradoxes of these arguments is that artificial hydration and nutrition are not considered LST, contravening well-established jurisprudential and medical-scientific positions.

In essence, the key element to qualify a LST, and consequently legitimize the AS, according to the CNB seems to be the *time* within which death occurs in its absence. Therefore, simple support is not sufficient because, upon its interruption, death—an event nonetheless expected—would not be quick or immediate, but would occur after an indefinable period, whereas the absence of the substitutive element results in rapid fatal effects. This is particularly peculiar considering that those who do not have the possibility of dying quickly would benefit from external assistance, in order to spare themselves and their loved ones further torment. This is even more true when considering the cornerstone of the legitimacy declaration sanctioned by the Constitutional Court: to ensure a self- determined process of death that is as rapid, painless, and dignified as possible, for patients who are now irreversible and irreparably suffering.

The majority opinion of the CNB was developed after a recent ECtHR ruling on an end-of-life case from Hungary. In this case, the ECtHR determined that there is no inherent right to AS under Article 8 of the European Convention on Human Rights (ECHR), nor could a discrimination claim be made under Article 14. The Court also highlighted the significance of existing safeguards in Hungary and other countries concerning the withdrawal of LST. It cautioned that providing physician-assisted suicide to individuals who do not meet the dependency criteria for LST could lead to further challenges and increase the risk of abuse 19.

When viewed outside the specific national contexts, this might appear as a significant retreat by the Strasbourg Court concerning the individual autonomy of patients at the end of life. However, it is precisely the ECtHR, which is determined to maintain the opportunity to issue a different judgment in the future “*considering the evolution of European societies and international standards of medical ethics in this sensitive area*,” that acknowledges its inability to surpass the recognized “*considerable margin of discretion*” of individual Member States, at least as long as many of them continue to “*criminalize such practices*.” Moreover, the “*highly delicate moral and ethical nature of the issue*,” the legal and legislative systems, and the domestic sociopolitical landscape require tailored and synchronous evaluative approaches by supranational bodies, constrained by an implicit limit on invasiveness and the protection of pluralism. Indeed, Member States retain the authority to “*continue to criminalize such practices and even prosecute individuals involved in these practices abroad against their own citizens.*”

Thus, mechanically transposing some of the ECtHR’s judgment passages into the current debate in Italy could lead to significant misunderstandings, given the clear disparity between the Hungarian legal system (where no form of physician-assisted suicide is permitted, and it is criminally prosecuted (Hungarian Act C of 2012 on the Criminal Code, 2012) even against foreign nationals for actions committed abroad (Author anonymous, 2025), with only refusal or cessation of life- sustaining treatments allowed for patients already in a terminal state) and the current Italian legal and normative framework.

Therefore, according to the Authors, the ECtHR ruling should not be interpreted as a judgment in favor of the indispensability of the DLST requirement, but rather as a legitimate endorsement of safeguard clauses and robust national regulatory frameworks to protect vulnerabilities, established with broad discretion by individual States. It should not be used to justify a substantial reversal of the current doctrine in Italy regarding end-of-life issues.

### 2.5 Sentence no. 135/2024 of the Italian Constitutional Court

In response to the Ordinance No. 32/2024, in sentence No. 135/2024 the Italian Constitutional Court has rejected the proposal to abolish the DLST requirement, deeming it not in conflict with the principles outlined by the Italian CC and the ECtHR: equality, therapeutic self-determination, human dignity, and respect for private life (Constitutional Court, 2024a).

The fundamental reason is essentially linked to the scope of Law no. 219 of 2017, which “in the absence of legislative intervention” remains an indispensable reference in assessing the legitimacy of AS. Thus, the rationale cannot be extended to patients who do not rely on LST, as they do not have the option to let themselves die—within a short period—simply by refusing care. The two situations were considered sufficiently different to invalidate the claim of unreasonable disparity in treatment of similar situations under Article 3 of the Constitution.

As acknowledged multiple times by the Constitutional Court, the 2019 declaration was closely tied to the absence of specific legislation on end-of-life issues, therefore being replaced by a law (No. 219/2017) which, as previously argued, has regulatory aims that pertain to a related but absolutely non- overlapping area to the one being examined.

It is interesting to note that in that evaluative context, the factor considered significant was the time within which death would occur following the cessation of treatment, comparing the circumstances of DJ Fabo’s case with other precedents (Constitutional Court, 2019), clearly alluding to the cases of E.E (Solarino et al., 2011). and P.W (Author anonymous, 2007). The latter was a patient suffering from muscular dystrophy who, no longer able to move and dependent on artificial ventilation via tracheostomy, repeatedly requested to end his life. His wish was eventually granted when he was assisted in dying through the administration of sedatives and the disconnection of the artificial respirator. It was tacitly admitted that P.W. could benefit from an almost immediate death by discontinuing the artificial ventilation; whereas the lower supportive intensity of DJ Fabo’s external ventilation would have forced him to achieve his suicidal goal after a longer period. This difference played a decisive role for the Court in judgment No. 242/2019, as in other cases, there is no room for judicial review of an explicit and declared request to die through the interruption of LST. Thus, in cases like that of DJ Fabo, the solution must be able to achieve the same right.

Hence, what is the name of this right? If its name is not the *right to die* (a term censured by the Court), it is nonetheless a form of liberation with lethal effects: it is not merely a refusal of therapies, but rather a refusal of certain forms of therapeutic interruption, to achieve a more direct terminal goal. Refusal of treatment and AS were considered equivalent to avoid imposing an unwanted or unreasonable solution.

On the other hand, under equal conditions (irreversible disease, source of intolerable suffering, with preserved capacity for self-determination), the legality of the external act ultimately depends on whether the person is kept alive by LST. Moreover, it is worth noting that the use of LST can arise from entirely accidental intrinsic and extrinsic variables: the diversity of concrete cases, the general clinical conditions of the patient (e.g., individual functionality and resistance of the body), manifestations of the disease (e.g., more or less advanced staging, more or less rapid progression), availability of therapies in a specific chronological and geographic context, personal decisions of individual patients (e.g., initial refusal of any treatment). The existence or non-existence of DLST appears entirely irrelevant and completely independent of, both abstractly and concretely, the presence of other requirements. It is widely accepted that, even from a medical perspective before a logical one, DLST does not imply the irreversibility of a disease and related suffering, nor is it sustainable to derive the existence of the former from the latter.

It is not clear how the requirement of DLST can be an appropriate and proportionate regulatory criterion to achieve the stated objective of safeguard for vulnerable individuals, protected by Article 580 of the Penal Code. As mentioned, it does not impact the indictability of assisting in dying (or, even more so, in extreme cases, the lesser need to protect these individuals); nor does it imply greater reassurance about the awareness of the choice or associate with any protective value on individual vulnerability. At most, it allows the opposite: a person dependent on LST and close to death, but not tempted to end their life, might be induced, even by external factors, to make this decision in a less than fully authentic manner. It is therefore not inconceivable to apply a parallel in the name of equality between patients meeting all four requirements indicated by the Court (which were equated with those who could already let themselves die by refusing LST) and those who at a certain point in their clinical history do not have the condition of DLST (such as terminally ill patients, those with cancer, or neurodegenerative diseases), often due to incidental causes, yet are similarly irreversible and forced to endure intolerable suffering, with the unreasonable exposure to longer periods of agony and pain that could be easily avoided.

Another aspect to consider is that prolonging the wait for death entails a greater burden of suffering and detriment to the person’s values, linked not only to the illness but also to the patient’s intentions to take this final step according to deeply personal convictions, driven also by the desire or interest to maintain a certain appearance of oneself or to depart from life following intimate views on the phenotypic expression of one’s personality. On the other hand, it is possible that patients who are subject to irreversible suffering, particularly those with a poor prognosis but not yet kept alive by medical treatments that align with the legal standards of DLST, may resort to autonomous solutions entirely devoid of the necessary legal safeguards.

The crucial innovation of the ruling is undoubtedly related to the expansion of the definition of DLST. The Court has consolidated the broader definition previously set by the Court of Massa, referring to procedures–even those not technically complex or highly invasive–generally deemed vital for the patient’s survival. This is a fundamental step since, unlike what the GIP of Florence suggested and also contradicting the CNB majority opinion, it confirms the possibility of including in the definition all those procedures that, “*regardless of the degree of technical complexity and invasiveness*”, (generally performed by healthcare personnel “*but that could be learned by patient’s relatives or caregivers*”) are “*concretely necessary to ensure the performance of vital functions of the patient, to the extent that their omission or interruption would likely lead to the patient’s death within a short period*” (8 of Considered in law). Examples include manual bowel evacuation, urinary catheterization, bronchial aspirations, artificial hydration, nutrition or ventilation. The thread connecting all these procedures, in the absence of a legislative definition, apparently be exposure to a ‘near death’ risk due to their refusal.

In any case, the requirement for LST must be maintained, as the Court believes it retains its protective value for situations of vulnerability and fragility that might otherwise be exposed to the risk of abuse or more or less direct coercion by third parties or arising from “*indirect social pressure*” (Constitutional Court, 2024b).

This is a strong, not insignificant stance, especially considering the decisions taken by the German, Austrian, and Spanish Constitutional Courts, from which the Italian Constitutional Court feels the need to diverge to reach a different evaluative outcome, given the distinct national context within which the current Italian legal landscape operates.

Nevertheless, the repeated references in the judgment to the current regulatory gap concerning end-of- life issues make it quite clear that the Court views its ruling not as a definitive solution but rather as a temporary palliative, pending clearer legislative guidance to address the now outdated void.

The need for a textual definition that overcomes the risk of often disparate and varied interpretations of individual rulings is undeniable. Such definitions should adhere strictly to regulatory provisions without interpretative licenses. The maximal extension of the requirement would include not only unexpected or underestimated situations but also those explicitly excluded by the Constitutional Court. Therefore, it is crucial to avoid the possibility of further semantic adjustments to the DLST requirement to prevent outcomes that are not only *praeter*, but even *contra legem*.

Another conquest obtained with Sentence No. 135/2024 is the admission of the requirement also when there are no LST in place because rejected by the patients themselves, based on a judgment of inappropriateness and overtreatment, according to Law No. 219/2017. Nevertheless, the need to maintain the DLST requirement, albeit in its extended definition, remains crucial for protecting vulnerable and fragile situations, which could otherwise be exposed to risks of abuse or direct or indirect persuasive pressures from third parties or social pressure. (7.2 of Considered in law).

Moreover, the most important aspect that we intend to emphasize is that the CC has repeatedly stressed that its role is not to replace the legislature in determining the balance between the right to self- determination and the right to life, but only to set the minimum constitutionally mandated guarantee, in light of the current legislative framework, leaving open a future legislative opportunity to find solutions that provide one or the other with more intense protection.

### 2.6 Tuscany Regional Law no. 16/2025

In the absence of action by the national legislature, the Tuscany region was the first (and currently only) region in Italy to clearly address the organizational procedures for accessing medically assisted suicide, by enacting Regional Law No. 16/2025. This law is in compliance with the conditions already established by the Constitutional Court’s rulings No. 242/2019 and No. 135/2024. The regional legislature’s initiative stems precisely from the need to remedy the persistent inaction of the Italian national legislature on the subject of physician-assisted suicide. However, the regional legislature is prevented from intervening on the specific requirements for accessing physician-assisted suicide, which is why the law refers back to the aforementioned Constitutional Court rulings. The law thus limits itself to regulating the procedures for accessing this process in its various phases. The law certainly represents a fundamental first step toward recognizing the right to self-determination at the end of life, while awaiting intervention from the national legislature to guarantee this right. Clearly, the law has sparked an intense political debate, and the matter has been brought before the Constitutional Court with an appeal filed by the government in May 2025. The appeal’s goal is to determine whether a region actually has the authority to regulate such a sensitive matter through a regional law.

## 3 Conclusion

It appears clear that the judgment invoked from the Constitutional Court, while admitting the broad definition of LST proposed by the Massese Court, primarily based on the thunderous parliamentary silence on end-of-life issues, has only minimally smoothed the harsh penal framework concerning AS, clarifying the unfounded nature of the potential constitutional issues raised by the various parties involved.

Regarding the existing Italian context, free from the specifics of the case examined by the Constitutional Court in 2019, it is particularly unacceptable that under equal conditions (irreversible disease, source of intolerable suffering, preserved capacity for self-determination), the access to AS ultimately depends on whether or not the person is kept alive by LST.

The existence or absence of DLST appears entirely irrelevant and completely independent of, both abstractly and concretely, the presence of other requirements. It is widely accepted that, even from a medical perspective before a logical one, DLST does not imply the irreversibility of a disease and related suffering, nor is it sustainable to derive the existence of the former from the latter.

It is therefore not inconceivable to apply a parallel in the name of equality, between patients meeting all four requirements indicated by the Court (which were equated with those who could already let themselves die by refusing LST) and those who at a certain point in their clinical history do not have the condition of DLST, often due to incidental causes, yet are similarly affected by irreversible diseases and forced to endure intolerable suffering, with the unreasonable exposure to longer periods of agony and pain that could be easily avoided.

However, given that the declaration is grounded in Law No. 219/17, the Court, while agreeing with many of the previously discussed criticisms, was unable to entirely dismantle the contested requirement, limiting itself to establishing the constitutional boundaries of social protection balancing them with individual rights and could not, in essence, override the significant margin of discretion afforded to the legislator, to whom it again defers the final decision.

At this point, one can only direct the question “*how much longer*?” to the legislator, as the path to take is absolutely clear: given the unfeasibility of a rational expansion of the current jurisprudential safeguard, streamlined by the clouded requirement of DLST, textual solutions must necessarily be sought. It is imperative that the legal framework on this matter not only results from adequate deliberation in the appropriate forums, according to the rules of constitutional democracy, but that, once established, it cannot be questioned through interpretative distortions by individual interpreters, which could lead to inconsistent and unpredictable applications that, even if favorable to the individual perpetrator, would undermine the deterrent and guiding function of the rule with respect to the protection of constitutionally defined essential values.

## Footnotes

1 It has been rightly noted by M. Massa in *“An Interlocutory Order on Assisted Suicide: Procedural Considerations at First Reading”*, published on www.forumcostituzionale.it, dated 01.12.2018, p. 4, that the four conditions outlined by the Court appear to be modeled after those indicated by the National Bioethics Committee for palliative deep sedation.

2 The accused individuals engaged in a series of actions that materially facilitated the execution of the self-destructive act: maintaining contact with the clinic; providing the necessary financial support to cover the costs of the procedure and travel; and alternating in driving the vehicle to the clinic. These actions collectively constituted necessary logical-causal antecedents—according to the conditionalist theory of causality—that made the suicide, as it ultimately occurred, possible. In their absence, the death would not have historically occurred in the described manner and timeframe.

## Abbreviations

PAS, physician-assisted suicide; LST, life-sustaining treatments; DLST, dependence on life-sustaining treatments LST; CC, Italian Constitutional Court; GIP, in Italian, judge for preliminary investigation; ECHR, European Convention on Human Rights; ECtHR, European Court on Human Rights; CNB, in Italian, national bioethics committee; ICC, Italian Criminal Code; EI, endotracheal intubation; PEG, percutaneous endoscopic gastrostomy.

## References

[B1] Author anonymous (2007). “Judge of the preliminary investigation of the court of rome,” in Judgment 23 July-17 October 2007. 2049.

[B2] Author anonymous (2024). European court of human rights, first section. Case of dániel karsai v. Hungary. 32312/23. Judgment 13/06/2024

[B3] Author anonymous (2025). Explanatory memorandum to the bill on the Hungarian criminal code: extraterritorial jurisdiction of the Hungarian criminal code.

[B4] Braun E. (2023). An autonomy-based approach to assisted suicide: a way to avoid the expressivist objection against assisted dying laws. J. Med. Ethics 49 (7), 497–501. 10.1136/jme-2022-108375 36190931 PMC10359509

[B5] Ciliberti R. Gorini I. Gazzaniga V. De Stefano F. Gulino M. (2018). The Italian law on informed consent and advance directives: new rules of conduct for the autonomy of doctors and patients in end-of-life care. J. Crit. Care 48, 178–182. 10.1016/j.jcrc.2018.08.039 30216936

[B6] Constitutional Court (2019). “Judgment n. 242 of 25th September 2019,” in Chapter 2.3 of considered in law.

[B7] Constitutional Court (2024a). “Judgment n. 135 of 18th July 2024,” in Chapter 7.1 of considered in law.

[B8] Constitutional Court (2024b). “Judgment n. 135 of 18th July 2024,” in Chapter 7.2 of considered in law.

[B9] Constitutional Court Press Office (2018). “Communication of 16 November 2018. Punishability of aid to suicide: to the legislator the regulation of limits,” in The Court's indications.

[B10] Corte D’Assise di Massa (2020). Judgment of 27/07/2020.

[B11] Cupelli C. (2020). The “Cappato case” and the lawful conducts of assisted suicide. From the 2 orders issued by the Italian constitutional court to the subsequent acquittal by assize court of milan. Cassaz. Penale 4, 1428.

[B12] Delbon P. Maghin F. Conti A. (2021). Medically assisted suicide in Italy: the recent judgment of the constitutional court. Clin. Ter. 172 (3), 193–196. 10.7417/CT.2021.2312 33956035

[B13] Di Fazio N. Romano S. Del Fante Z. Santoro P. Fineschi V. Frati P. (2021). European countries' different legal orientation about end-of-life issues in patients affected with neurological/psychiatric diseases: does Italian law n.219/2017 provide adequate options for this fragile category of patients? Front. Psychiatry 12, 675706. 10.3389/fpsyt.2021.675706 34630172 PMC8497821

[B14] Dyer O. White C. García Rada A. (2015). Assisted dying: law and practice around the world. BMJ 351, h4481. 10.1136/bmj.h4481 26290517

[B15] Emanuel E. J. Onwuteaka-Philipsen B. D. Urwin J. W. Cohen J. (2016). Attitudes and practices of euthanasia and Physician- assisted suicide in the United States, Canada, and Europe. JAMA 316 (1), 79–90. 10.1001/jama.2016.8499 27380345

[B16] Gazzetta Ufficiale (2024). Judge of the preliminary investigation of the court of florence. Order n.32 of 17 January 2024. Gazzetta Uff. 11. Available online at: https://i2.res.24o.it/pdf2010/S24/Documenti/2024/03/15/AllegatiPDF/Ordinanza%20rimessione.pdf.

[B17] Hungarian Act C of 2012 on the Criminal Code (2012). Section 162: aiding and abetting suicide.

[B18] Jox R. J. (2023). Of slopes and ropes: learning from the diversity of European regulations of assisted dying. Am. J. Bioeth. 23 (11), 84–87. 10.1080/15265161.2023.2256281 37879028

[B19] Kitzinger J. Kitzinger C. (2017). Why futile and unwanted treatment continues for some PVS patients (and what to do about it). Case study, context and policy recommendations. Int. J. Ment. Health Capacity Law 129, 84–149.

[B20] Kon A. A. Shepard E. K. Sederstrom N. O. Swoboda S. M. Marshall M. F. Birriel B. (2016). Defining futile and potentially inappropriate interventions: a policy statement from the society of critical care medicine ethics committee. Crit. Care Med. 44, 1769–1774. 10.1097/CCM.0000000000001965 27525995

[B21] Marrone M. Berardi P. Solarino B. Ferorelli D. Corradi S. Silvestre M. (2022). Italian legal euthanasia: unconstitutionality of the referendum and analysis of the “Italian” problem. Front. Sociol. 7, 898783. 10.3389/fsoc.2022.898783 35903266 PMC9315237

[B22] Montanari V. G. (2019). The marco cappato and fabiano antoniani (dj fabo) case paves the way for new assisted suicide legislation in Italy: an overview of statutes from several European countries. Eur. J. Health Law 26 (3), 221–239. 10.1163/15718093-12261428 31220807

[B23] Mroz S. Dierickx S. Deliens L. Cohen J. Chambaere K. (2021). Assisted dying around the world: a status quaestionis. Ann. Palliat. Med. 10 (3), 3540–3553. 10.21037/apm-20-637 32921084

[B24] National Bioethics Committee (CNB) (2023). Response of 20.6.2024 to the question of the territorial ethics committee of the Umbria region of 3, 11.

[B25] Ricci G. Gibelli F. Sirignano A. (2022). Editorial - from ruling no. 242/2019 of the constitutional court to the Italian law on medically assisted death: a complex transition. Eur. Rev. Med. Pharmacol. Sci. 26 (13), 4546–4549. 10.26355/eurrev_202207_29174 35856342

[B26] Sallnow L. Smith R. Ahmedzai S. H. Bhadelia A. Chamberlain C. Cong Y. (2022). Report of the lancet commission on the value of death: bringing death back into life. Lancet 399 (10327), 837–884. 10.1016/S0140-6736(21)02314-X 35114146 PMC8803389

[B27] Savulescu J. (2002). Is there a “right not to be born”? Reproductive decision making, options and the right to information. J. Med. Ethics 28, 65–67. 10.1136/jme.28.2.65 11934928 PMC1733557

[B28] Solarino B. Bruno F. Frati G. Dell'erba A. Frati P. (2011). A national survey of Italian physicians' attitudes towards end-of-life decisions following the death of eluana englaro. Intensive Care Med. 37 (3), 542–549. 10.1007/s00134-011-2132-5 21287145

[B29] Turillazzi E. Maiese A. Frati P. Scopetti M. Di Paolo M. (2021). Physician-patient relationship, assisted suicide and the Italian constitutional court. J. Bioeth. Inq. 18 (4), 671–681. 10.1007/s11673-021-10136-w 34674155

[B30] van Wijngaarden E. Leget C. Goossensen A. (2015). Ready to give up on life: the lived experience of elderly people who feel life is completed and no longer worth living. Soc. Sci. Med. 138, 257–264. 10.1016/j.socscimed.2015.05.015 25982088

