# Correction: The night is dark and full of hesitation. The latest step in the legal trajectory of assisted suicide in Italy: through tentative judicial openings, bioethical contradictions and the Parliament’s deafness

**DOI:** 10.3389/fphar.2026.1808055

**Published:** 2026-02-25

**Authors:** Frontiers Production Office

**Affiliations:** Frontiers Media SA, Lausanne, Switzerland

**Keywords:** medical aid in dying, voluntary assisted death, assisted suicide, end-of-life, right to self-determination, life-sustaining treatments, constitutional legitimacy

To credit Dr. Marietjie Wilhelmina Maria Botes’ contribution to the initial stages of this article’s peer-review process, the following Acknowledgements statement has been added to the published article:

“Dr. Marietjie Wilhelmina Maria Botes oversaw the initial stages of this article’s peer-review process as the handling editor.”

The original article has been updated.

